# Effect of aromatic substituents on thermoresponsive functional polycaprolactone micellar carriers for doxorubicin delivery

**DOI:** 10.3389/fphar.2024.1356639

**Published:** 2024-03-04

**Authors:** Hanghang Wang, Himanshu Polara, Abhi Bhadran, Tejas Shah, Godwin Kweku Babanyinah, Ziyuan Ma, Erika L. Calubaquib, Justin T. Miller, Michael C. Biewer, Mihaela C. Stefan

**Affiliations:** Department of Chemistry and Biochemistry, University of Texas at Dallas, Richardson, TX, United States

**Keywords:** functionalized polycaprolactone, amphiphilic diblock copolymers, thermoresponsive polymers, polymeric micelles, drug delivery

## Abstract

Amphiphilic functional polycaprolactone (PCL) diblock copolymers are excellent candidates for micellar drug delivery. The functional groups on the backbone significantly affect the properties of PCL. A systematic investigation of the effect of aromatic substituents on the self-assembly of γ-functionalized PCLs and the delivery of doxorubicin (DOX) is presented in this work. Three thermoresponsive amphiphilic diblock copolymers with poly(γ-benzyloxy-ε-caprolactone) (PBnCL), poly(γ-phenyl- ε-caprolactone) (PPhCL), poly(γ-(4-ethoxyphenyl)-ε-caprolactone) (PEtOPhCL), respectively, as hydrophobic block and γ-tri(ethylene glycol) functionalized PCL (PME_3_CL) as hydrophilic block were prepared through ring-opening polymerization (ROP). The thermoresponsivity, thermodynamic stability, micelle size, morphology, DOX-loading, and release profile were determined. The LCST values of amphiphilic diblock copolymers PME_3_CL-*b*-PBnCL, PME_3_CL-*b*-PPhCL, and PME_3_CL-*b*-PEtOPhCL are 74.2°C, 43.3°C, and 37.3°C, respectively. All three copolymers formed spherical micelles in phosphate-buffered saline (PBS, 1×, pH = 7.4) at low concentrations ranging from 8.7 × 10^−4^ g/L to 8.9 × 10^−4^ g/L. PME_3_CL-*b*-PBnCL micelles showed the highest DOX loading capacity of 3.01 ± 0.18 (wt%) and the lowest drug release, while PME_3_CL-*b*-PEtOPhCL micelles exhibited the lowest DOX loading capacity of 1.95 ± 0.05 (wt%) and the highest drug release. Cytotoxicity and cellular uptake of all three micelles were assessed *in vitro* using MDA-MB-231 breast cancer cells. All three empty micelles did not show significant toxicity to the cells at concentrations high up to 0.5 mg/mL. All three DOX-loaded micelles were uptaken into the cells, and DOX was internalized into the nucleus of the cells.

## Graphical Abstract

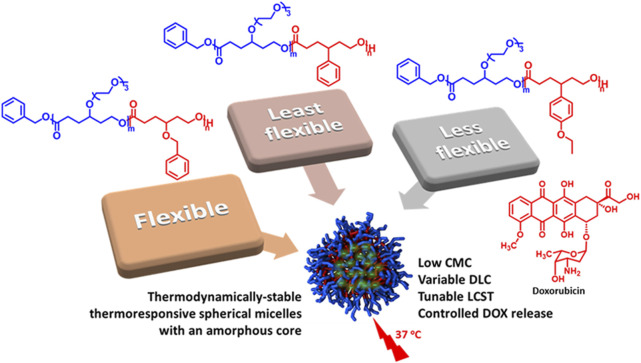

## 1 Introduction

Polymeric micelles formed from polycaprolactone (PCL)-based amphiphilic diblock copolymers can be employed in drug delivery applications due to their biocompatibility and (bio)degradability. (Shah and Vasava, 2019; Ahmad Shariff et al., 2022; Kuperkar et al., 2022; Bhadran et al., 2023a; Patha et al., 2023). The micelles can physically pack poorly water-soluble drugs in their hydrophobic core and stabilize them in aqueous solutions with the help of their hydrophilic shell. The functional groups on the PCL diblock copolymers can be varied to adjust the properties of polymeric micelles, including hydrophilicity, thermodynamic stability, drug loading capacity, and site targeting. (Bhadran et al., 2023b).

The hydrophilicity of polymeric micelles is important for drug delivery because it allows the micelles to solubilize in the bloodstream and reduces reticuloendothelial system clearance. (Wang et al., 2023). Hydrophilic functional groups such as *N*-isopropylamide (Dai et al., 2008) and oligo(ethylene glycol) (OEG) (Soltantabar et al., 2020; Calubaquib et al., 2021) were reported to provide hydrophilicity to PCL-based polymers. Dai et al. (Dai et al., 2008) compared the water absorbance rate of a *N*-isopropylamide functionalized PCL copolymer and an unfunctionalized PCL polymer. The functionalized PCL bearing 9.1 mol% *N*-isopropyl-2-carbamoylethyl group absorbed 43.2 wt% water after 70 days, more than 80 times the water absorbed by the unfunctionalized PCL. However, no micelle preparation was performed. Calubaquib et al. (Calubaquib et al., 2021) synthesized OEG functionalized PCL homopolymers and obtained thermoresponsive polymeric micelles with lower critical solution temperature (LCST) in water. The OEG groups contributed hydrophilicity and thermoresponsivity to the micelles, and those properties were easily tuned by varying the length of the OEG groups. It is beneficial to use thermoresponsive micelles for drug delivery, considering the potential enhancement of therapeutic efficacy by thermal-controlled drug release. The desired LCST of thermoresponsive micelles is slightly above the body temperature. Unfortunately, the OEG functionalized PCL homopolymers (>58°C) are unsuitable for drug delivery due to undesired high LCST. (Calubaquib et al., 2021). Soltantabar and Calubaquib et al. (Soltantabar et al., 2020) also reported thermosensitive polymeric micelles prepared by OEG-functionalized PCL copolymers in water. The LCST was tuned to 15°C–59°C by adding a hydrophobic block, and thermal-controlled release of the anticancer drug doxorubicin was achieved. (Soltantabar et al., 2020).

Thermodynamic stability is also of great value when selecting suitable polymeric micelles for drug delivery because they should maintain their form to prevent premature drug release upon dilution after intravenous injection into the bloodstream. The desired CMC is below10^−3^ g/L, (Rainbolt et al., 2015a), and it can be tuned by varying the functional groups on the copolymer (Hao et al., 2013; Washington et al., 2018a) and by adjusting the ratio of the two blocks of amphiphilic diblock copolymers. (Hao et al., 2011; Rainbolt et al., 2015b; Senevirathne et al., 2015; Senevirathne et al., 2017a).

Drug loading capacity (DLC) is another essential consideration for micellar drug delivery. Higher drug loading capacities reduce the number of carrier materials for a given drug dosage, improving production and administration and mitigating concerns about the pharmacological effect of the carrier material. DLC can be affected by the hydrophobic functional groups on the polymers. (Lee et al., 2010; Yan et al., 2010; Hao et al., 2011; Liu et al., 2011; Yan et al., 2011; Hao et al., 2013; Huang et al., 2013; Peng et al., 2013; Shi et al., 2013; Zhang et al., 2014; Rainbolt et al., 2015b; Glavas et al., 2015; Senevirathne et al., 2015; Malhotra et al., 2016; Surnar and Jayakannan, 2016; Senevirathne et al., 2017a; Washington et al., 2018a; Qiao et al., 2020; Wen et al., 2020). This is attributed to the interactions between the drug payload and the hydrophobic micellar core. (Takahashi et al., 2014). A practical approach to increasing the DLC of drugs containing aromatic rings is to enhance the π-π stacking interaction between the drug payload and the hydrophobic micellar core. (Takahashi et al., 2014). Yan et al. (Yan et al., 2011) reported carbamic acid benzyl ester (CAB) functionalized PCL-based diblock copolymers for the delivery of doxorubicin (DOX). The DLC of the copolymers increased from 19.0% to 25.5% with the increase in the content of the CAB group. Peng et al. (Peng et al., 2013) obtained a higher DLC of indomethacin, a nonsteroidal anti-inflammable drug, using phenyl functionalized PCL-based diblock copolymers *versus* unfunctionalized PCL copolymers. Washington et al. (Washington et al., 2018a) investigated the DOX loading capacity of benzyloxy functionalized PCL-based diblock copolymer, which was 2.2 times more than that of the unfunctionalized PCL copolymers. The high DLC of functionalized PCL copolymers was attributed to the enhanced π-π stacking interactions between DOX and the hydrophobic benzyloxy functional groups on the copolymers.

As mentioned earlier, PCL-based amphiphilic diblock copolymers bearing aromatic functional groups can increase the DLC of micelles. However, to our knowledge, a systematic comparison of the influence of analogous aromatic functional groups on micellization, thermodynamic stability, drug loading, release, and cytotoxicity has yet to be studied. Therefore, we systematically investigated the effect of three analogous aromatic substituents on the self-assembly behaviors of γ-functionalized PCL diblock copolymers and the delivery of DOX. Tri(ethylene glycol) functionalized PCL (PME_3_CL) was used as a hydrophilic block, and γ-aromatic group functionalized PCLs bearing benzyloxy, phenyl, and 4-ethoxyphenyl groups were utilized as hydrophobic blocks. The tri(ethylene glycol) (ME_3_) functional group was used to provide hydrophilicity and thermoresponsivity to the diblock copolymers. The aromatic functional groups are significantly different in flexibility, and it decreased in the order of benzyloxy (Bn) > 4-ethoxyphenyl (EtOPh) > phenyl (Ph). The effect of the flexibility and steric of the hydrophobic aromatic substituents on the critical micellar concentration (CMC), LCST, DOX loading, and release was assessed. *In vitro* studies of cytotoxicity and cellular uptake were carried out on MDA-MB-231 breast cancer cells.

## 2 Materials and methods

### 2.1 Materials

All chemicals were obtained from Sigma-Aldrich or Fisher Scientific. Prior to use, benzyl alcohol (BnOH) was purified by vacuum distillation. Chloroform was dried over calcium hydride (CaH_2_) and purified by vacuum distillation. All glassware used for polymerizations was kept in an oven heated at 120°C for 24 h and cooled down in a desiccator before use. The monomers were dried with CaH_2_ before polymerization. The ring-opening polymerization (ROP) was performed in a glovebox at room temperature. The hydrophilic monomer γ-tri(ethylene glycol) ε-caprolactone (γ-ME_3_CL) (Hao et al., 2013; Soltantabar et al., 2020) and the hydrophobic monomers γ-benzyloxy ε-caprolactone (γ-BnCL) (Hao et al., 2013; Soltantabar et al., 2020) and γ-phenyl ε-caprolactone (γ-PhCL) (Deng et al., 2001) were synthesized following the reported procedure in the literature. The new hydrophobic monomer γ-4-ethoxyphenyl ε-caprolactone (γ-EtOPhCL) was prepared following a two-step synthetic route (Supplementary Scheme S1).

### 2.2 Analysis

A Bruker AVANCE III (500 MHz) nuclear magnetic resonance (NMR) instrument was used to collect ^1^H and ^13^C spectra using CDCl_3_ as the solvent. Size exclusion chromatography (SEC) measurements were obtained using a Shimadzu HPLC instrument equipped with an Agilent column connected to the Shimadzu refractive index detector with N, N-dimethylformamide (DMF) as eluent, and poly(methyl methacrylate) (PMMA) standard calibration. Differential scanning calorimetry (DSC) was performed on a TA Instruments Q100 DSC under nitrogen at 20 mL/min. A temperature-controlled Cary5000 UV-vis spectrometer was used for the turbidimetric assay of the synthesized polymers. A 0.5°C min^-1^ heating rate was applied, and a quartz cuvette with a path length of 1.0 cm was used. Fluorescence spectroscopy was performed using a Perkin-Elmer LS 50 BL luminescence spectrometer. The size and distribution of the particles were measured through dynamic light scattering (DLS) using the Malvern Zetasizer Nano ZS instrument equipped with a He-Ne laser (633 nm) and a 173° backscatter detector. Transmission electron microscopy (TEM) analysis was conducted using a JEM-1400+ TEM (JEOL United States Inc., MA) with 2% phosphotungstic acid stain. A Biotek Cytation 3 imaging reader was used to perform cell viability and cellular uptake measurements.

### 2.3 Synthesis of polymers

#### 2.3.1 Synthesis of diblock copolymer poly{γ-2-[2-(2-methoxyethoxy)ethoxy]ethoxy-ε-caprolactone}-*b*-poly(γ-benzyloxy-ε-caprolactone) (PME_3_CL-*b*-PBnCL)

In a 10 mL round bottom flask, organocatalyst triazabicylo[4.4.0]dec-5-ene (TBD) (5.57 mg, 0.04 mmol) and benzyl alcohol (BnOH) initiator (4.33 mg, 0.04 mmol) in dry chloroform were stirred for 30 min. Then, the hydrophilic γ-ME_3_CL monomer (552.66 mg, 2.00 mmol) was added to the flask. After complete conversion, the hydrophobic γ-BnCL monomer (440.54 mg, 2.00 mmol) was added. The reaction was stirred till the second monomer was fully converted. Then the polymerization was quenched by exposing it to air and adding acetic acid. The polymer was obtained by precipitation in hexane/THF. ^1^H NMR (500 MHz, CDCl_3_): δ 1.76–1.95 (m, 8H), 2.31–2.46 (m, 4H), 3.37 (s, 3H), 3.45–3.72 (m, 14H), 4.09–4.21 (m, 4H), 4.42–4.55 (m, 2H), 7.30–7.38 (m, 5H). ^13^C NMR (500 MHz, CDCl_3_): δ 28.79, 29.09, 29.68, 29.76, 32.85, 33.11, 59.02, 61.26, 61.33, 68.54, 70.55, 70.63, 70.72, 71.08, 71.95, 74.60, 74.65, 75.80, 127.67, 127.78, 128.39, 138.29, 173.39, 173.39, 173.44. Mn = 5600, PDI = 1.67.

#### 2.3.2 Synthesis of diblock copolymer poly{γ-2-[2-(2-methoxyethoxy)ethoxy]ethoxy-ε-caprolactone}-*b*-poly(γ-phenyl-ε-caprolactone) (PME_3_CL-*b*-PPhCL)

A similar method was used to synthesize amphiphilic diblock copolymer PME_3_CL-*b*-PPhCL: ^1^H NMR (500 MHz, CDCl_3_): δ 1.76–1.97 (m, 8H), 1.99–2.04 (m, 2H), 2.37–2.41 (m, 2H), 2.54–2.58 (q, 1H), 3.37 (s, 3H), 3.44–3.48 (q, 1H), 3.53–3.65 (m, 12H), 3.77–3.88 (m, 2H), 4.15–4.18 (t, 2H), 7.02–7.22 (m, 5H). ^13^C NMR (500 MHz, CDCl_3_): δ 29.09, 29.76, 31.60, 32.09, 32.11, 33.11, 35.39, 42.18, 59.02, 61.32, 62.63, 68.54, 70.56, 70.63, 70.73, 71.95, 75.79, 126.68, 127.56, 128.63, 143.01, 173.23, 173.44. Mn = 9600, PDI = 1.95.

#### 2.3.3 Synthesis of diblock copolymer poly{γ-2-[2-(2-methoxyethoxy)ethoxy]ethoxy-ε-caprolactone}-*b*-poly(γ-(4-ethoxylphenyl)-ε-caprolactone) (PME_3_CL-*b*-PEtOPhCL)

A similar method was used to synthesize amphiphilic diblock copolymer PME_3_CL-*b*-PEtOPhCL: ^1^H NMR (500 MHz, CDCl_3_): δ 1.38–1.41 (t, 3H), 1.77–1.90 (m, 8H), 1.99–2.01 (m, 2H), 2.37–2.43 (m, 2H), 2.48–2.57 (m, 1H), 3.37 (s, 3H), 3.44–3.49 (m, 1H), 3.53–3.65 (m, 12H), 3.73–3.87 (m, 2H), 3.96–3.99 (t, 2H), 4.15–4.17 (t, 2H), 6.79–6.^13^C NMR (500 MHz, CDCl_3_): δ 14.90, 29.09, 29.74, 31.78, 32.17, 33.10, 35.54, 41.29, 59.03, 61.32, 62.67, 63.36, 68.54, 70.56, 70.63, 70.72, 71.95, 75.78, 114.59, 128.41, 134.75, 157.66, 173.36, 173.45. Mn = 9300, PDI = 1.95.

### 2.4 Determination of CMC

The CMC was determined by using pyrene as a hydrophobic fluorescence probe. A series of aqueous polymer solutions in phosphate-buffered saline (PBS, 1×, pH = 7.4) with varying polymer concentrations and a constant pyrene concentration were prepared using THF stock solutions of polymers and pyrene. THF was removed by vigorous stirring and evaporation. The final polymer concentrations were 10^−8^ to 1 g/L, and the final pyrene concentration was 6.55 × 10^−7^ M. Then, the pyrene-loaded micelle aqueous solutions were subjected to a fluorescence spectrometer to collect fluorescence excitation spectra (emission at 390 nm).

### 2.5 Determination of LCST

The LCST was determined by using a temperature-controlled UV-Vis spectrometer. Polymers were directly dissolved in phosphate-buffered saline (PBS, 1×, pH = 7.4) and equilibrated for 24 h. Then, the aqueous polymer solutions (1 mg/mL) were subjected to a temperature-controlled UV-Vis spectrometer to record transmittance with temperature change.

### 2.6 Micelle preparation

Empty micelles were prepared by the solvent evaporation method. A 1.5 mL THF polymer solution was added dropwise to 5 mL phosphate-buffered saline (PBS, 1×, pH = 7.4) while homogenizing for 15 min. Then, the THF was evaporated by vortex, and micelle solutions were obtained by filtration using a 0.45 µm nylon filter. The final polymer concentration was 1 mg/mL.

### 2.7 Drug loading

DOX-loaded micelles were prepared using the solvent evaporation method. DOX^.^ HCl solution was neutralized by adding three equiv. of triethylamine in THF. To 5 mL phosphate-buffered saline (PBS, 1×, pH = 7.4), a 1.5 mL THF solution of 5 mg polymer and 0.5 mg DOX was added dropwise with homogenization for 15 min. THF was removed by vortex, and DOX-loaded micelle solution (1 mg/mL) was obtained by filtration using a 0.45 µm nylon filter. To determine drug loading capacity (DLC) and drug loading efficiency (DLE), the DOX-loaded micelles were first disassembled by DMSO and then subjected to a UV-Vis spectrometer to record absorbance spectra. The concentration of DOX was determined by fitting absorbance readout at 485 nm to a pre-plotted absorbance vs. DOX concentration in DMSO:PBS (1:1) calibration curve. DLC and DLE were calculated using the equations listed below.

$$\text{DLC}=\frac{mass\;of\;Dox\;loaded}{mass\;of\;total\;polymer}\times 100\%$$ (1)

$$\text{DLE}=\frac{mass\;of\;Dox\;loaded}{mass\;of\;total\;Dox}\times 100\%$$ (2)

### 2.8 Size and morphology analysis

The micelle size was measured by dynamic light scattering (DLS). After micelles preparation in PBS with a concentration of 1 mg/mL, samples were equilibrated at 25°C in a Malvern Zetasizer Nano ZS instrument to measure the hydrodynamic diameters (D_h_) of micelles. Morphological studies of the micelle solution were carried out by Transmission Electron Microscopy (TEM). 2 wt% phosphotungstic acid (PTA) solution was used to stain the micelles to analyze the morphology.

### 2.9 Determination of *in vitro* drug release

Freshly prepared DOX-loaded micelles with a polymer-to-DOX feed ratio of 10:1 were used to determine the release profile at various conditions. The loaded micelle solution (4 mL), with a constant concentration of polymer at 1 mg/mL, was transferred to a SnakeSkin^®^ dialysis tubing with a MW cutoff of 3500 Da and was dialyzed against 10 mL of PBS at a pH of 7.4 for 48 h at 37°C. At specific intervals, 0.3 mL of the release media was removed and replaced with fresh PBS. Each sample was diluted with DMSO and analyzed to quantify the amount of DOX released based on the pre-established calibration curve using UV−Vis spectroscopy. Absorbance measurements in the release media were taken at 485 nm to calculate the cumulative DOX release.

### 2.10 Biological studies

Unless otherwise indicated, all cell culture experiments were performed using Dulbecco’s Modified Eagle Medium (DMEM) medium with 10% fetal bovine serum (FBS) and 1% penicillin–streptomycin. Cells were grown in a humidified environment at 37°C with 5% CO_2_.

#### 2.10.1 Cytotoxicity studies

MDA-MB-231 Breast cancer (ATCC, HTB-26) cells were cultured in DMEM supplemented with 10% FBS and 1% penicillin/streptomycin (pen/strep). The cells were seeded in a 96-well plate at a density of 1 × 10^4^ cells per well in 100 μL growth medium. Cells were allowed to adhere to the bottom of the wells by incubation at 37°C at 5% CO_2_ for 24 h. The medium was then removed, and the cells were washed with PBS. A total of 80 μL of DOX-loaded micelle (in PBS) of different concentrations were added to each well, followed by an additional 100 μL of fresh media. After 24 h of incubation at 37°C, the media containing drug-loaded micelles were removed, and the cells were washed twice using PBS. The cells were treated with 30 μL of 12 mM methylthiazolyldiphenyl-tetrazolium bromide (MTT) (Thermo Fisher Scientific, United States of America) in PBS and incubated for 5 h at 37°C and 5% CO_2_ in the dark to form formazan crystals. The formazans were dissolved using 170 μL of dimethyl sulfoxide (DMSO). The absorption was recorded at 540 nm and normalized to the intensity of the untreated cells (N = 5, standard deviation). Statistical analysis was performed with one-way ANOVA using GraphPad Prism. The value of *p* < 0.05 was considered statistically significant.

#### 2.10.2 Cellular uptake

MBA-MB-231 cells were cultured in a 24-well plate at a density of 2 × 10^4^ cells per well. The cells were treated with 0.5 mg/mL (100 μL) DOX-loaded micelle along with 200 μL fresh DMEM media and further incubated at 37°C in 5% CO_2_ for 4 h. After the incubation, the media was removed, and the cells were washed with PBS three times. The cells were then fixed with 4% paraformaldehyde (incubation for 10 min at room temperature) and washed with PBS (300 μL × 2). After that, the nucleus was counter-stained with 4′,6-diamidino-2-phenylindole (DAPI, blue) according to the manufacturer’s recommended protocol. The images were taken using the BioTek Cytation 3 fluorescent microscope to determine the cellular uptake of micelles.

## 3 Result and discussion

### 3.1 Synthesis of amphiphilic diblock copolymers

**FIGURE 1:**
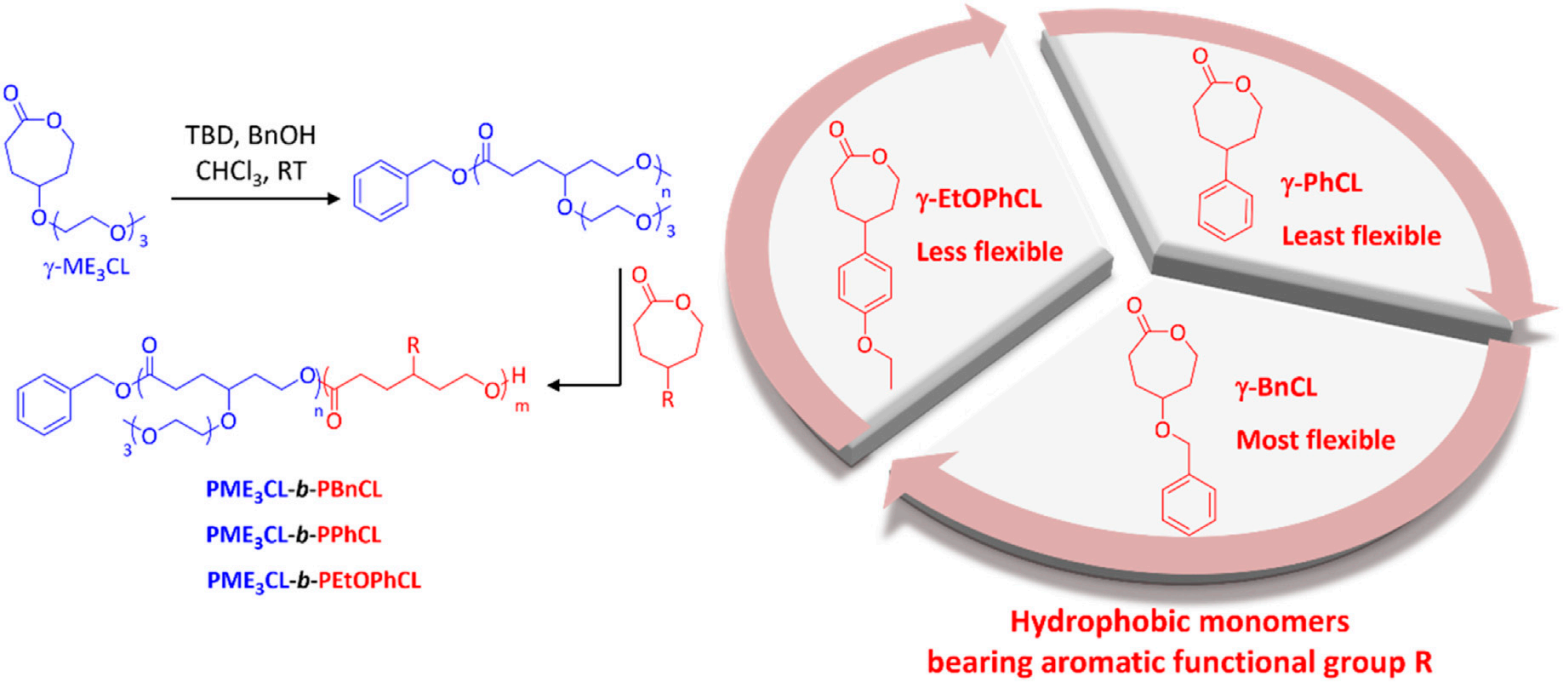
Synthesis of amphiphilic diblock copolymers poly{γ-2-[2-(2-methoxyethoxy)ethoxy]ethoxy-ε-caprolactone}-*b*-poly(γ-benzyloxy-ε-caprolactone) (PME_3_CL-*b*-PBnCL), poly{γ-2-[2-(2-methoxyethoxy)ethoxy]ethoxy-ε-caprolactone}-*b*-poly(γ-phenyl-ε-caprolactone) (PME_3_CL-*b*-PPhCL), and poly{γ-2-[2-(2-methoxyethoxy)ethoxy]ethoxy-ε-caprolactone}-*b*-poly(γ-(4-ethoxylphenyl)-ε-caprolactone) (PME_3_CL-*b*-PEtOPhCL). TBD: triazabicylo[4.4.0]dec-5-ene. BnOH: benzyl alcohol.

Amphiphilic diblock copolymers were prepared by sequential monomer addition through ROP using organocatalysts TBD and BnOH initiator (Figure 1). The hydrophilic block is comprised of γ-ME_3_CL monomer. The hydrophilic ME_3_ substituents contributed to the thermoresponsivity of the amphiphilic diblock copolymers and, therefore, to the micelles. (Hao et al., 2011; Cheng et al., 2012; Hao et al., 2013; Rainbolt et al., 2015b; Soltantabar et al., 2020; Calubaquib et al., 2021). The hydrophobic blocks consist of three different γ-aromatic group functionalized ε-caprolactone monomers with variable flexibility (Figure 1). The flexibility of the hydrophobic monomers decreases in the order of γ-BnCL > γ-EtOPhCL > γ-PhCL. The monomers γ-ME_3_CL, (Hao et al., 2013; Soltantabar et al., 2020), γ-BnCL, (Hao et al., 2013; Soltantabar et al., 2020), and γ-PhCL (Deng et al., 2001) were synthesized according to literature. The new monomer γ-EtOPhCL was synthesized by reacting 4-(4-hydroxyphenyl)cyclohexan-1-one with bromoethane and potassium carbonate to produce 4-(4-ethoxyphenyl)cyclohexan-1-one, which was then purified using column chromatography. Treatment with 77% *m*CPPBA was subjected to Baeyer–Villiger oxidation to form γ-4-ethoxyphenyl ε-caprolactone (γ-EtOPhCL). The structure of γ-EtOPhCL was characterized by ^1^H and ^13^C NMR (Supplementary Figures S1–S7). The structure of this monomer was further confirmed by GC-MS analysis. (Supplementary Figure S8).

The synthesized amphiphilic diblock copolymers were characterized by ^1^H and ^13^C NMR analysis (Supplementary Figures S9–S14). The composition and molecular mass of the copolymers are summarized in Table 1. The estimated molecular mass from ^1^H NMR were calculated by multiplying the degree of polymerization (DP_n_) of polymers with the molecular mass of monomers and then adding the molecular mass of the initiator. The DP_n_ of PME_3_CL-*b*-PBnCL was determined by the ratio of the integrations of the methoxy protons of ME_3_ group on the hydrophilic block (∼3.4 ppm), the methylene protons of benzyl substituent on the hydrophobic block (∼4.5 ppm), and the methylene protons of the benzyl end group of the initiator (∼5.1 ppm). The DP_n_ of PME_3_CL-*b*-PPhCL and PME_3_CL-*b*-PEtOPhCL were determined by the ratio of integrations of the methoxy protons of ME_3_ group on the hydrophilic block, the proton of the tertiary carbon on the hydrophobic block backbone (∼2.5 ppm), and the methylene protons of the benzyl end group of the initiator.

**TABLE 1:** Molecular mass and components of synthesized amphiphilic diblock copolymers.

Polymer	Feed ratio (mol%)	Measured ratio^a^ (mol%)	M_n_ ^a^ (kDa)	M_n_ ^b^ (kDa)	PDI^b^ (M_w_/M_n_)
PME_3_CL-*b*-PBnCL	50:50	46:54	35.7	5.6	1.67
PME_3_CL-*b*-PPhCL	50:50	45:55	25.4	9.6	1.95
PME_3_CL-*b*-PEtOPhCL	50:50	47:53	36.6	9.3	1.95

^a^ Calculated by ^1^H NMR spectra.

^b^ Determined by size exclusion chromatography (SEC).

The molecular mass determined by size exclusion chromatography (SEC) (Supplementary Figure S15) are lower than those estimated by ^1^H NMR. This discrepancy may arise from differences in the hydrodynamic volumes of the polymers and the poly(methyl methacrylate) standard used for calibration. The polydispersity index (PDI) of the synthesized diblock copolymers varies from 1.67–1.95. Differential scanning calorimetry (DSC) suggests all three diblock copolymers are amorphous (Supplementary Figures S16). The glass transition temperature (T_g_) of the hydrophilic block PME_3_CL is −80°C, comparable to the reported −82°C. (Hao et al., 2013). The T_g_ of hydrophobic block PBnCL is −51°C, slightly lower than those of PPhCL (−47°C) and PEtOPhCL (−48°C). This can be attributed to the difference of flexibility and chemical structures among these polymers. The benzyl group of PBnCL is attached to PCL backbone with an ether linkage, while the phenyl group of PPhCL and 4-ethoxyphenyl group of PEtOPhCL are directly connected to the PCL backbone.

### 3.2 Determination of thermoresponsivity (LCST)

**FIGURE 2:**
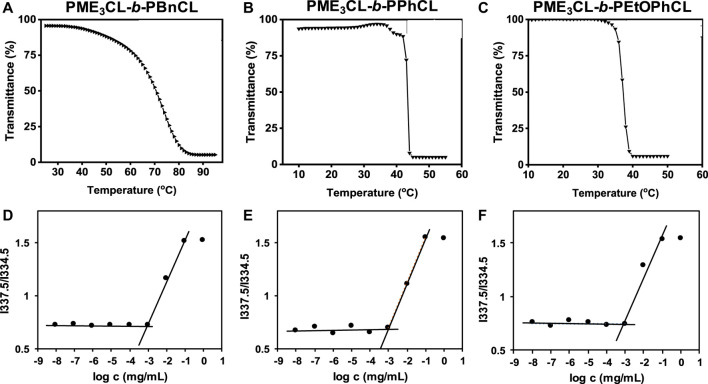
Thermoresponsivity **(A–C)** and thermodynamic stability **(D–F)** of diblock copolymers (1 mg/mL polymer aqueous solutions in PBS (1×, pH = 7.4)). CMC was determined using pyrene as a hydrophobic fluorescence probe.

Functional polycaprolactone polymers containing ME_3_ substituents were reported to have lower critical solution temperature (LCST), above which the polymers underwent phase transition to become less soluble in aqueous solutions. (Hao et al., 2011; Hao et al., 2013; Rainbolt et al., 2015b; Soltantabar et al., 2020; Calubaquib et al., 2021). Temperature-controlled UV-Vis spectrometer is commonly utilized to reveal the thermoresponsivity of the synthesized diblock copolymers. The LCST is taken as the temperature at which the transmittance drops 50%. (Soltantabar et al., 2020; Calubaquib et al., 2021; Ghasemi et al., 2022). After the direct dissolution of polymers in PBS (1×, pH = 7.4) (1 mg/mL) and equilibration for 24 h, the aqueous polymer solutions were taken into a temperature-controlled UV-Vis spectrometer. The LCST values of PME_3_CL-*b*-PBnCL, PME_3_CL-*b*-PPhCL, and PME_3_CL-*b*-PEtOPhCL are 74.2°C, 43.3°C, and 37.3°C, respectively (Figures 2A–C). Notably, PME_3_CL-*b*-PPhCL and PME_3_CL-*b*-PEtOPhCL bearing less flexible aromatic substituents exhibited shaper phase transition and lower LCST. The lowest LCST of PME_3_CL-*b*-PEtOPhCL is attributed to the highest hydrophobicity of the polymer. (Hao et al., 2011; Rainbolt et al., 2015b).

### 3.3 Determination of thermodynamic stability (CMC)

Amphiphilic diblock copolymers can self-assemble in aqueous solutions above their CMC to form core-shell structured micelles. (Hussein and Youssry, 2018; Li and Lin, 2022). The CMC is an important parameter to determine the thermodynamic stability of polymeric micelles for drug delivery applications because the micelles with higher CMC values disassemble immediately upon substantial dilution after intravenous injection into the bloodstream. (Rainbolt et al., 2015a; Perumal et al., 2022). Empty micelles were prepared by a solvent evaporation method. Briefly, a polymer solution in THF was added dropwise to PBS (1×, pH = 7.4) with homogenization. Empty micelle aqueous solutions were obtained after evaporation of THF by vortex and filtration with a 0.45 µm nylon filter. The CMC of PME_3_CL-*b*-PBnCL, PME_3_CL-*b*-PPhCL, and PME_3_CL-*b*-PEtOPhCL were determined using pyrene as a hydrophobic fluorescence probe. Aqueous polymer solutions in PBS with varying polymer concentrations and constant pyrene were analyzed by fluorescence spectrometer. The abrupt change in the ratio of fluorescence intensities at wavelengths of 337.5 nm and 334.5 nm was used to determine the CMC. The CMC values of PME_3_CL-*b*-PBnCL, PME_3_CL-*b*-PPhCL, and PME_3_CL-*b*-PEtOPhCL are 8.8 × 10^−4^ g/L, 8.7 × 10^−4^ g/L, and 8.9 × 10^−4^ g/L, respectively (Figures 2D–F). There is no significant difference in CMC values of the synthesized diblock copolymers due to their similar chemical structures. However, the lower CMC values imply that the self-assembled micelles are stable even at lower concentrations.

### 3.4 Size and morphology of micelles

**FIGURE 3:**
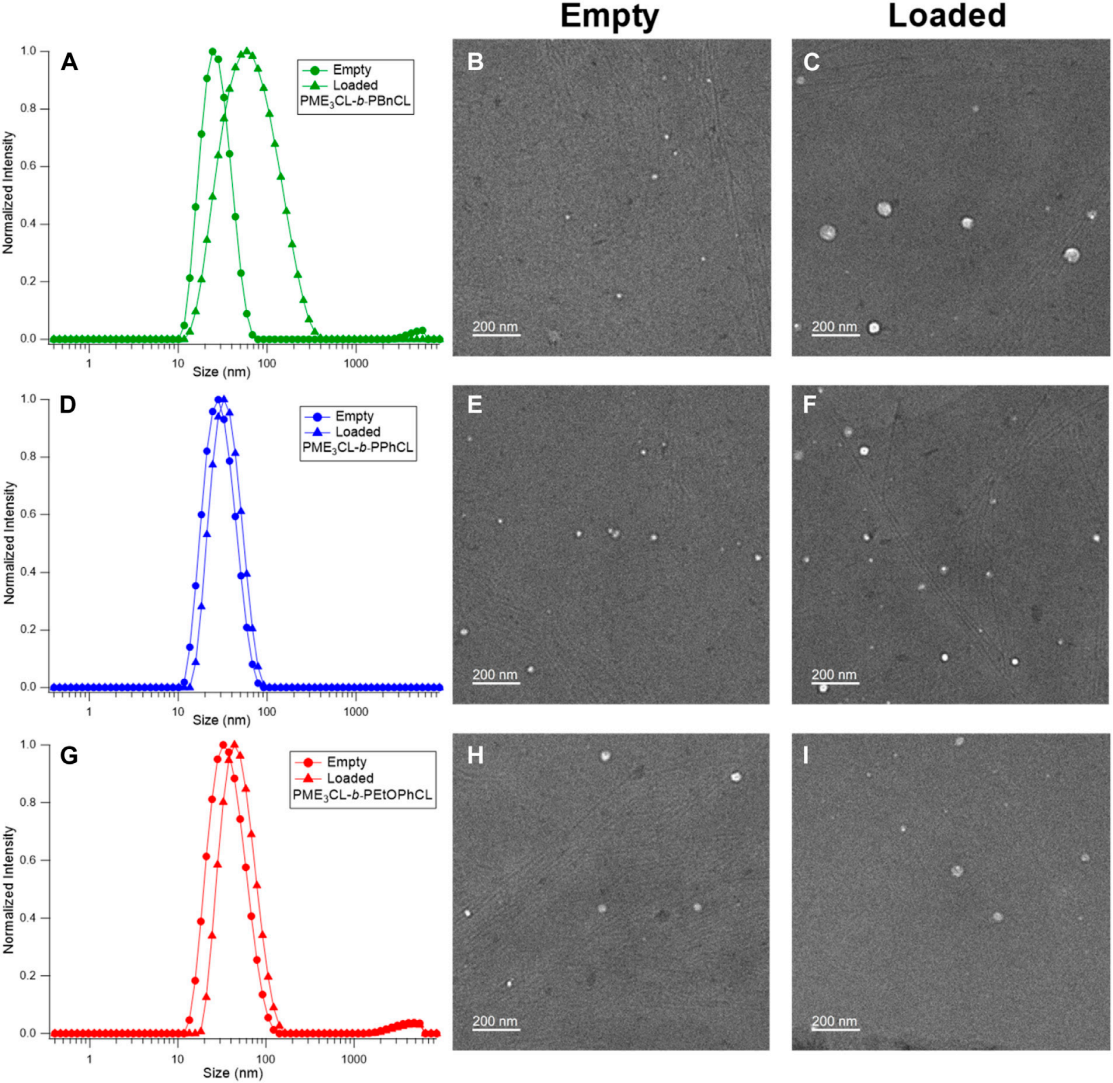
DLS and TEM of empty micelles and DOX-loaded micelles of PME_3_CL-*b*-PBnCL **(A–C)**, PME_3_CL-*b*-PPhCL **(D–F)** and PME_3_CL-*b*-PEtOPhCL **(G–I)**.

The empty micelle sizes were determined by DLS (Figures 3A, D, G), which are 25.30 nm, 26.94 nm, and 34.15 nm for PME_3_CL-*b*-PBnCL, PME_3_CL-*b*-PPhCL, and PME_3_CL-*b*-PEtOPhCL, respectively (Table 2). The empty micelle sizes increase in the order of PME_3_CL-*b*-PBnCL, PME_3_CL-*b*-PPhCL, and PME_3_CL-*b*-PEtOPhCL. This is expected since the more flexible benzyl functional groups arrange themselves to form compact micelles. Compared to the most rigid phenyl group, the less rigid 4-ethyoxyphenyl group is more sterically hindered due to the additional ethoxy group at the para-position, which may further hinder the arrangement of the 4-ethoxyphenyl groups in micellar core, resulting in larger micelles. TEM images showed the formation of spherical micelles and smaller sizes (Figures 3B, E, H). This is because micelles get dehydrated when preparing TEM samples.

**TABLE 2:** Properties of empty and DOX-loaded micelles.

Polymer	Size in nm (dispersity) *^a^*		DLC (wt%)	DLE (wt%)
	Empty micelles	DOX-loaded micelles		
PME_3_CL-*b*-PBnCL	25.30 (0.155)	49.76 (0.292)	3.01 ± 0.18	30.10 ± 1.88
PME_3_CL-*b*-PPhCL	26.94 (0.118)	31.96 (0.116)	2.31 ± 0.25	23.10 ± 2.55
PME_3_CL-*b*-PEtOPhCL	34.15 (0.196)	45.48 (0.173)	1.95 ± 0.05	19.50 ± 0.50

^a^ DLS, was used to determine the hydrodynamic diameter and size dispersity.

### 3.5 Drug loading with doxorubicin

The potential application of the micelles as drug carriers was tested by loading DOX, an anticancer hydrophobic drug molecule. (Chen et al., 2015; Rainbolt et al., 2015b; Malhotra et al., 2016; Senevirathne et al., 2017b; Washington et al., 2017; Washington et al., 2018b; Chuan et al., 2019; Soltantabar et al., 2020; Yan et al., 2020; Karmegam et al., 2021; Song et al., 2021). DOX-loaded micelles were prepared by entrapping the drug in the hydrophobic core using the solvent evaporation method. Similarly, a solution of polymer and DOX in THF was added to the aqueous solution dropwise while homogenizing. DOX-loaded micelle solutions were obtained after filtration by a 0.45 µm nylon filter. Upon loading of DOX, the size of micelles was slightly increased (Figures 3A, D, G). The sizes of DOX-loaded micelles were 49.76 nm, 31.96 nm, and 45.48 nm for PME_3_CL-*b*-PBnCL, PME_3_CL-*b*-PPhCL, and PME_3_CL-*b*-PEtOPhCL, respectively (Table 2). TEM images demonstrated the formation of spherical DOX-loaded micelles (Figures 3C, F, I).

The drug loading capacity of DOX-loaded micelles was determined by UV-Vis spectroscopy. Briefly, DOX-loaded micelles were disassembled by adding an equivalent volume of DMSO, and then the UV-Vis absorbance of the mixture was obtained. The concentration of DOX was determined by fitting the absorbance readings to a pre-plotted calibration curve. Then, the DLC and DLE were calculated using Eqs. 1, 2. PME_3_CL-*b*-PBnCL exhibited the highest DLC, while PME_3_CL-*b*-PPhCL and PME_3_CL-*b*-PEtOPhCL showed lower and comparable DLC. This can be attributed to the flexibility of the aromatic groups on the hydrophobic blocks. Due to higher flexibility, the benzyloxy groups on the PBnCL block can orient themselves to accommodate DOX molecules and enhance the polymer-drug interaction, which is impossible in the other two polymers. The DLC of PEtOPhCL is hampered by considerable steric hindrance of the 4-ethoxy group on the phenyl ring. This limits the polymers’ interaction with DOX, resulting in a low loading of DOX into the PME_3_CL-*b*-PEtOPhCL micellar core.

### 3.6 Drug release profile

**FIGURE 4:**
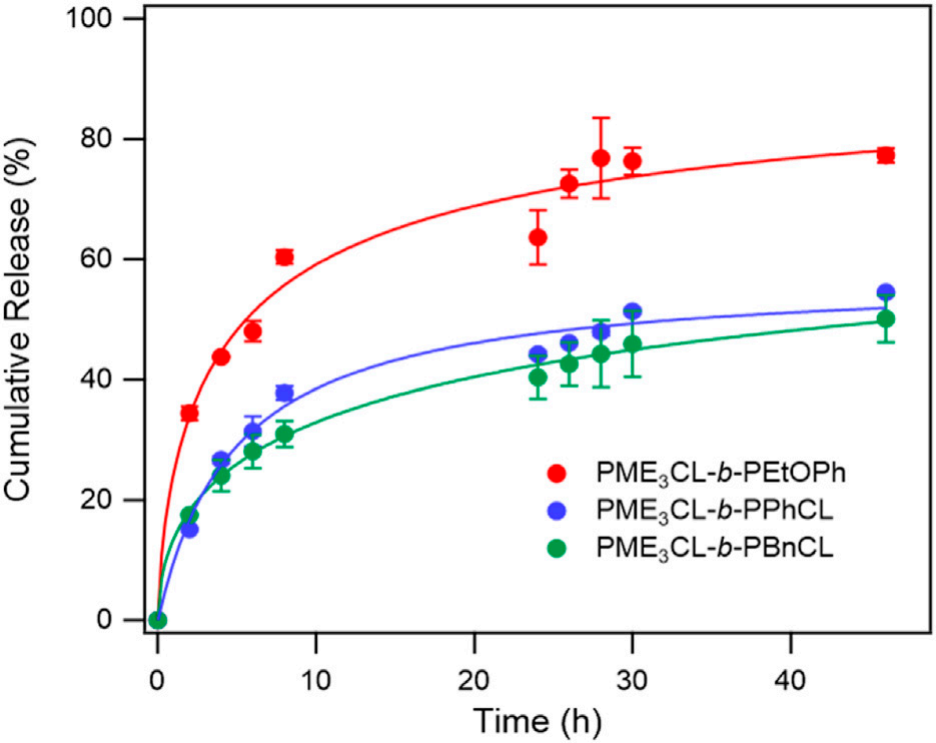
Release profiles of DOX from DOX-loaded micelles at physiological temperature in PBS buffer solution (pH 7.4), N = 3.

The *in vitro* release of DOX from the micelles was studied in PBS (pH 7.4) at a physiological temperature (37°C) to compare the effect of the hydrophobic aromatic side chain (Figure 4). The initial release rate of PME_3_CL-*b*-PEtOPhCL micelles was higher than that of the PME_3_CL-*b*-PBnCL and PME_3_CL-*b*-PPhCL micelles, which confirms that the polymer with a lower LCST shows a faster release profile. The overall release of the PME_3_CL-*b*-PEtOPhCL micelles was the highest, achieving a cumulative release of 60% after 8 h. This is attributed to its LCST being closest to the physiological temperature. (Soltantabar et al., 2020; Dou et al., 2023). The release study indicates that the DOX release of the reported thermoresponsive polymeric micelles is affected by their LCST values and that the drug release can be adjusted by varying the hydrophobic aromatic functional groups on the polymers.

### 3.7 Toxicity assessment

**FIGURE 5:**
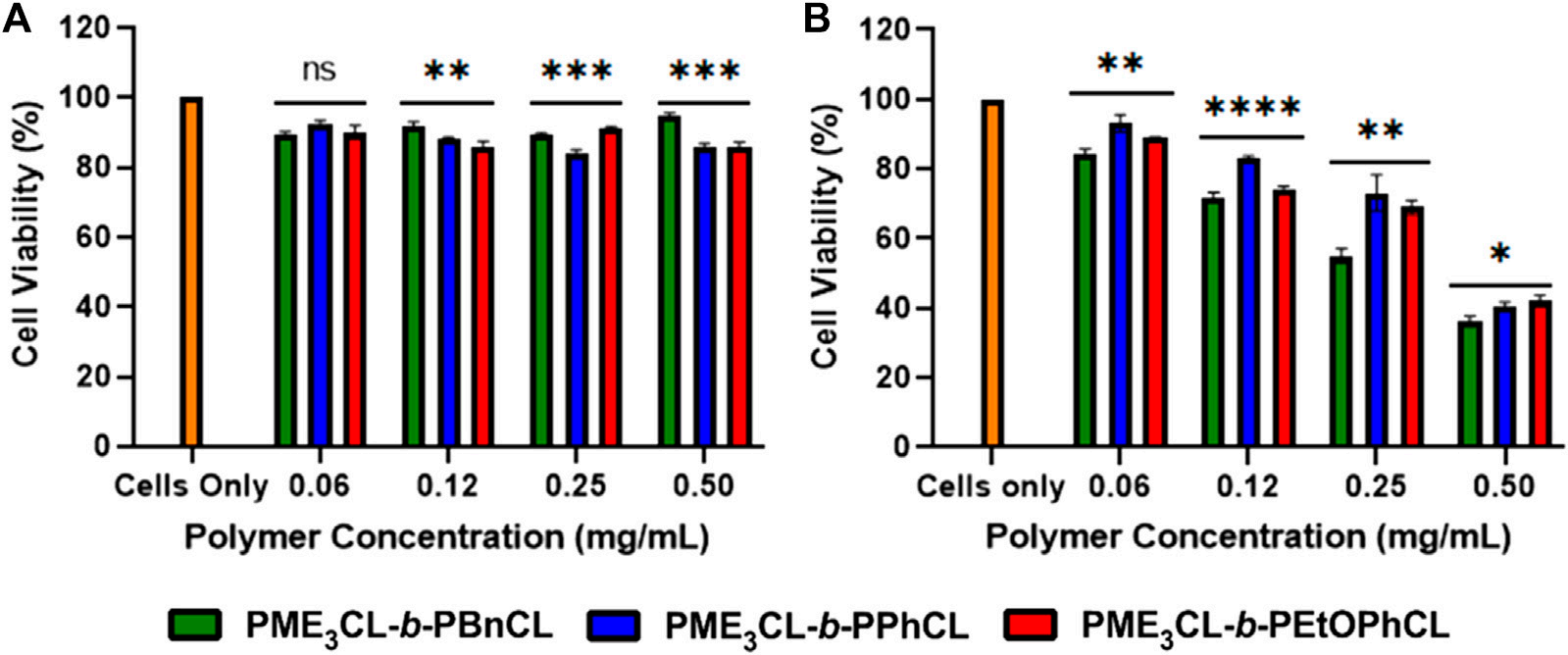
Cell viability measurements by varying concentrations of **(A)** empty polymeric micelles **(B)** DOX-loaded micelles using MBA-MB-231 cells, N = 5.

Cytotoxicity measurements were conducted on MDA-MB-231 breast cancer cells using various concentrations of empty micelles to assess polymer biocompatibility. The cell viability was examined by MTT assay, followed by the cells had been exposed to the polymer solutions for 24 h. The empty micelles were not shown to exhibit significant toxicity to the cells even up to 0.5 mg/mL (Figure 5A). The cytotoxicity measurements demonstrate that polymers are excellent biocompatible materials for drug delivery applications.

To test the cytotoxicity of DOX-loaded micelles, the micelles were loaded into MDA-MB-231 cells and incubated for 24 h under 37°C and 5% CO_2._ It was observed at all concentrations that the release of DOX was more significant to the drug loading capacity at physiological temperatures. The DOX-loaded PME_3_CL-*b*-PBnCL micelles exhibited higher toxicity than the DOX-loaded PME_3_CL-*b*-PEtOPhCL micelles, which can be attributed to the increased loading capabilities of the PME_3_CL-*b*-PBnCL micelles (Figure 5B).

### 3.8 Cellular uptake

**FIGURE 6:**
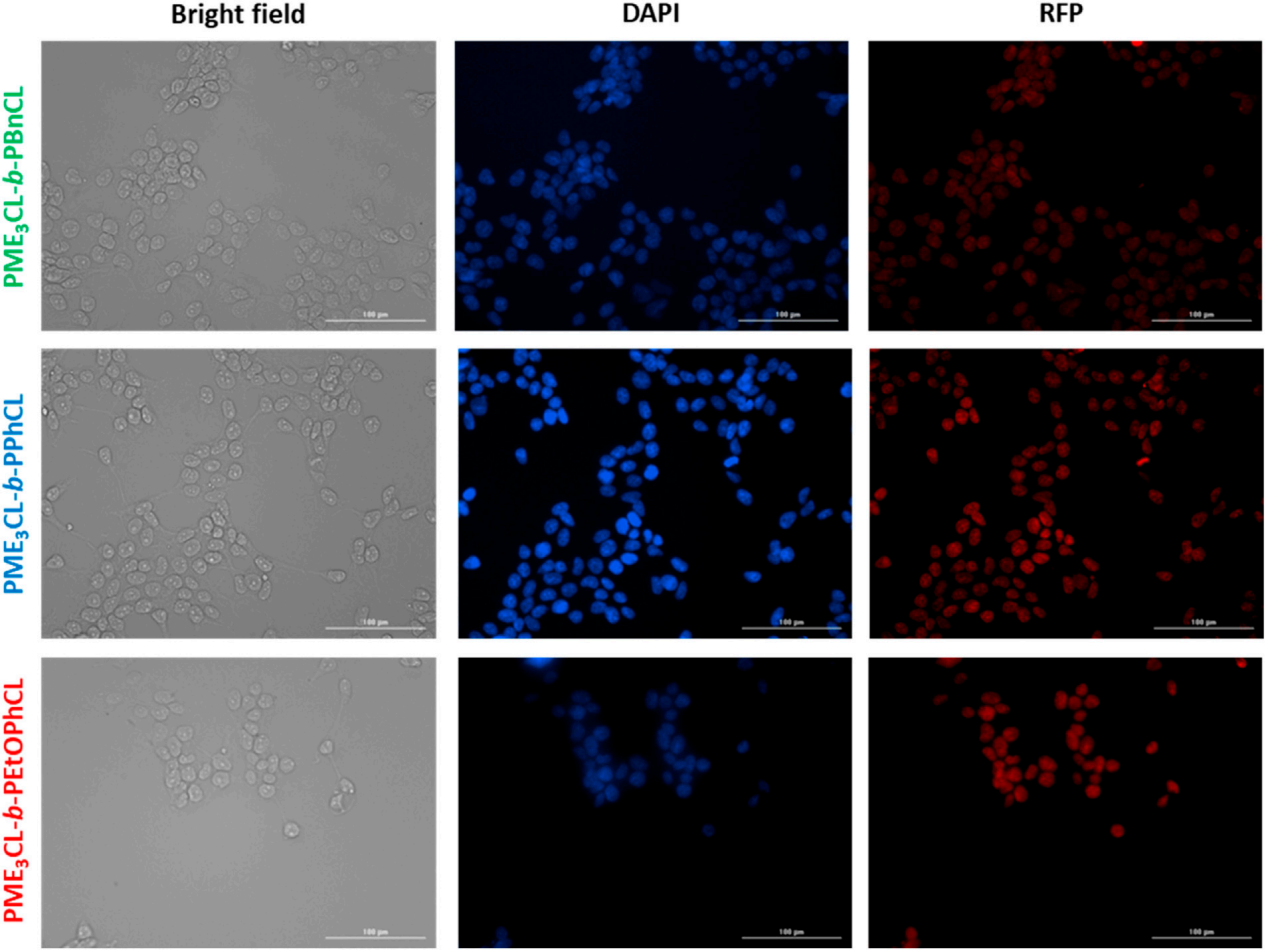
Cellular uptake of DOX-loaded micelles into MDA-MB-231 cells at 37°C. Fluorescent images were recorded after 4 h of incubation. Images from left to right show bright field, cells with nuclei staining using DAPI, and imaged with RFP filter. Scale bar = 100 μm.

Cellular uptake of DOX-loaded micelles was performed using MDA-MB-231 cells at 37°C. The cells were introduced with the DOX-loaded micelles and incubated for 4 h. After fixing the cells, the cell nuclei were stained with DAPI and visualized through Cytation 3 fluorescent microscope (Figure 6). The data reveals that the DOX-loaded micelles were preferentially accumulated into the cells, and the DOX were internalized within the nucleus of the cells. Moreover, the cells incubated with PME_3_CL-*b*-PEtOPhCL showed a slightly higher DOX internalization into the nucleus when compared to the cells incubated with PME_3_CL-*b*-PBnCL and PME_3_CL-*b*-PPhCL at 37°C. This further confirms that the micelles with higher LCST values were less uptaken than those with the closest LCST to the physiological temperature. The red signal, attributed to DOX, can be seen within the cell nuclei, indicating the endocytosis of the micelles into the cell and the internalization of DOX into the nucleus of the cell.

## 4 Conclusion

Three *γ*−functionalized PCL amphiphilic diblock copolymers with varying hydrophobic aromatic substituents (PME_3_CL-*b*-PBnCL, PME_3_CL-*b*-PPhCL, and PME_3_CL-*b*-PEtOPhCL) were synthesized. All three diblock copolymers are amorphous and can form thermodynamically stable spherical micelles with comparable CMC. Their potential usage as drug carriers was assessed by loading with DOX. PME_3_CL-*b*-PBnCL exhibited the highest DLC, while PME_3_CL-*b*-PPhCL and PME_3_CL-*b*-PEtOPhCL showed lower DLC due to higher structural rigidity and larger steric hindrance. However, the LCST values of these two polymers were closer to physiological temperature, and their DOX-loaded micelles exhibited faster drug release. All three amphiphilic diblock copolymers were shown to have minimal toxicity on MDA-MB-231 breast cancer cells up to a concentration of 0.5 mg/mL. *In vitro* studies also confirmed that the cells were less viable at higher concentrations of DOX-loaded micelles. In this work, a systematic study of the effect of analogous aromatic functional groups on the self-assembly behaviors of γ-functionalized PCL amphiphilic diblock copolymers and the delivery of anticancer drug DOX was reported. In the future, we plan to investigate the influence of different linkages (such as ether, ester, and amide), which connect the functional groups and the polymer backbone, on the performance of amphiphilic functional PCL micellar carriers for the delivery of anticancer drugs.

## Data Availability

The original contributions presented in the study are included in the article/Supplementary Material, further inquiries can be directed to the corresponding authors.
