# Supplementary material for: Effect of aromatic substituents on thermoresponsive functional polycaprolactone micellar carriers for doxorubicin delivery
Source: Front Pharmacol. 2024 Mar 4;15:1356639. doi: 10.3389/fphar.2024.1356639 (PMC10945023; doi:10.3389/fphar.2024.1356639)
Supplement: Supplementary file 1 [file DataSheet1.docx]

Effect of Aromatic Substituents on Thermoresponsive Functional Polycaprolactone Micellar Carriers for Doxorubicin Delivery

Hanghang Wang^†^, Himanshu Polara^†^, Abhi Bhadran, Tejas Shah, Godwin Kweku Babanyinah, Ziyuan Ma, Erika L. Calubaquib, Justin T. Miller, Michael C. Biewer*, Mihaela C. Stefan*

Department of Chemistry and Biochemistry, University of Texas at Dallas, Richardson, TX, USA

*Correspondence to Mihaela C. Stefan: [mihaela@utdallas.edu](mailto:mihaela@utdallas.edu) and Michael C. Biewer [biewerm@utdallas.edu](mailto:biewerm@utdallas.edu)

^†^ These authors contributed equally to this work.

**Supporting Information**

**Fig. S1**. ^1^H NMR spectrum of intermediate compound 4-(4-ethoxyphenyl)cyclohexan-1-one

**Fig. S2**. ^13^C NMR spectrum of intermediate compound 4-(4-ethoxyphenyl)cyclohexan-1-one

**Fig. S3**. ^13^C DEPT 135 NMR spectrum of intermediate compound 4-(4-ethoxyphenyl)cyclohexan-1-one

**Fig. S4**. ^1^H NMR spectrum of monomer γ-4-ethoxyphenyl ε-caprolactone (γ-EtOPhCL)

**Fig. S5**. ^13^C NMR spectrum of monomer γ-4-ethoxyphenyl ε-caprolactone (γ-EtOPhCL)

**Fig. S6**. ^13^C DEPT 135 NMR spectrum of mononer γ-4-ethoxyphenyl ε-caprolactone (γ-EtOPhCL)

**Fig. S7**. HSQC NMR spectrum of monomer γ-4-ethoxyphenyl ε-caprolactone (γ-EtOPhCL)

**Fig. S8**. GC-MS of monomer γ-4-ethoxyphenyl ε-caprolactone (γ-EtOPhCL)

**Fig S9**. ^1^H NMR spectrum of amphiphilic diblock poly{γ-2-[2-(2-methoxyethoxy)ethoxy]ethoxy-ε-caprolactone}-*b*-poly(γ-benzyloxy-ε-caprolactone) (PME_3_CL-*b*-PBnCL)

**Fig S10**. ^13^C NMR spectrum of amphiphilic diblock poly{γ-2-[2-(2-methoxyethoxy)ethoxy]ethoxy-ε-caprolactone}-*b*-poly(γ-benzyloxy-ε-caprolactone) (PME_3_CL-*b*-PBnCL)

**Fig. S11**. ^1^H NMR spectrum of amphiphilic diblock copolymer poly{γ-2-[2-(2-methoxyethoxy)ethoxy]ethoxy-ε-caprolactone}-*b*-poly(γ-phenyl-ε-caprolactone) (PME_3_CL-*b*-PPhCL)

**Fig. S12**. ^13^C NMR spectrum of amphiphilic diblock copolymer poly{γ-2-[2-(2-methoxyethoxy)ethoxy]ethoxy-ε-caprolactone}-*b*-poly(γ-phenyl-ε-caprolactone) (PME_3_CL-*b*-PPhCL)

**Fig. S13**. ^1^H NMR spectrum of amphiphilic diblock copolymer poly{γ-2-[2-(2-methoxyethoxy)ethoxy]ethoxy-ε-caprolactone}-*b*-poly(γ-(*4*-ethoxylphenyl)-ε-caprolactone) (PME_3_CL-*b*-PEtOPhCL)

**Fig. S14**. ^13^C NMR spectrum of amphiphilic diblock copolymer poly{γ-2-[2-(2-methoxyethoxy)ethoxy]ethoxy-ε-caprolactone}-*b*-poly(γ-(*4*-ethoxylphenyl)-ε-caprolactone) (PME_3_CL-*b*-PEtOPhCL)

**Fig. S15**. GPC spectra of amphiphilic diblock copolymers PME_3_CL-*b*-PBnCL, PME_3_CL-*b*-PPhCL, and PME_3_CL-*b*-PEtOPhCL.

**Fig. S16**. DSC of amphiphilic diblock copolymers PME_3_CL-*b*-PBnCL, PME_3_CL-*b*-PPhCL, and PME_3_CL-*b*-PEtOPhCL.

**Monomer Synthesis**

**Scheme S1**. Synthesis of hydrophobic γ-(4-ethoxyphenyl)-ε-caprolactone monomer (γ-EtOPhCL).

Synthesis of 4-(4-ethoxyphenyl)cyclohexan-1-one. 4-(4-Hydroxyphenyl)cyclohexan-1-one (3.8 g, 0.02 mol), bromoethane ( 23.97 g, 0.22 mol), and potassium carbonate ( 5.5 g, 0.04 mol) were added to a round bottom flask along with acetone as solvent. The reaction mixture was refluxed overnight and then filtered to remove the undissolved solid. The solvent was removed under vacuum, and column chromatography (hexane : ethyl acetate = 10:1 ) was used to obtain pure product 4-(4-ethoxyphenyl)cyclohexan-1-one (white solid: 3.75 g, 86%). 1H NMR (500 MHz, CDCl3): δ 1.39-1.42 (t, 3H), 1.86-1.95 (m, 2H), 2.17-2.22 (m, 2H), 2.45-2.54 (m, 4H), 2.94-3.01 (tt, 1H), 3.99-4.04 (q, 2H), 6.84-6.87 (m, 2H), 7.13-7.16 (m, 2H). 13C NMR (500 MHz, CDCl_3_): δ 14.89, 34.21, 41.41, 41.92, 63.44, 114.55, 127.54, 136.75, 157.59, 211.30.

Synthesis of 5-(4-ethoxyphenyl)oxepan-2-one (γ-EtOPhCL). 4-(4-ethoxyphenyl)cyclohexan-1-one (3.75 g, 0.017 mol) and 77% mCPPBA (5.8 g, 0.026mol) were stirred in dichloromethane overnight and then filtered to remove undissolved solid. K_2_CO_3_ was used to remove excess mCPBA. After filteration and removing solvent under vacuum, the crude product was recrystallized in hexane : ethyl acetate (2 : 1) to obtain pure monomer 5-(4-ethoxyphenyl)oxepan-2-one (γ-EtOPhCL) (white solid, 3.2 g, 80%). 1H NMR (500 MHz, CDCl_3_): δ 1.39-1.42 (t, 3H), 1.76-1.84 (m, H), 1.95-2.13 (m, H), 2.71-2.82 (m, H), 3.99-4.03 (q, H), 4.27-4.31 (t, 1H), 4.36-4.40 (dddd, 1H), 6.83-6.85 (d, 2H), 7.07-7.09 (d, 2H). 13C NMR (500 MHz, CDCl3): δ 14.86, 30.60, 33.69, 37.00, 46.41, 63.47, 68.26, 114.68, 127.50, 137.05, 157.77, 175.70.


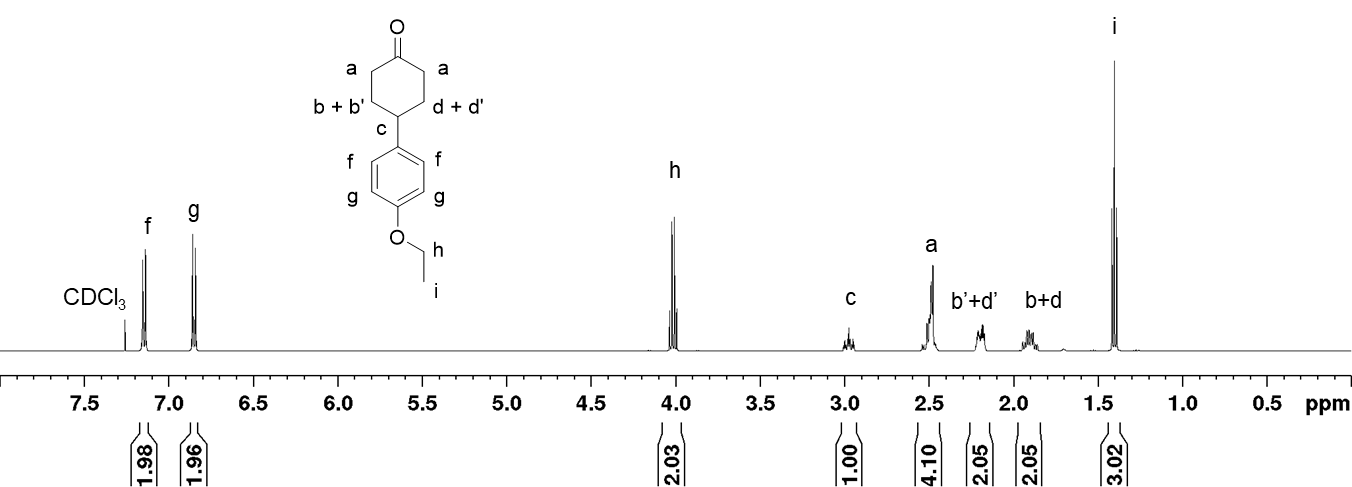


**Fig. S1**. ^1^H NMR spectrum of intermediate compound 4-(4-ethoxyphenyl)cyclohexan-1-one


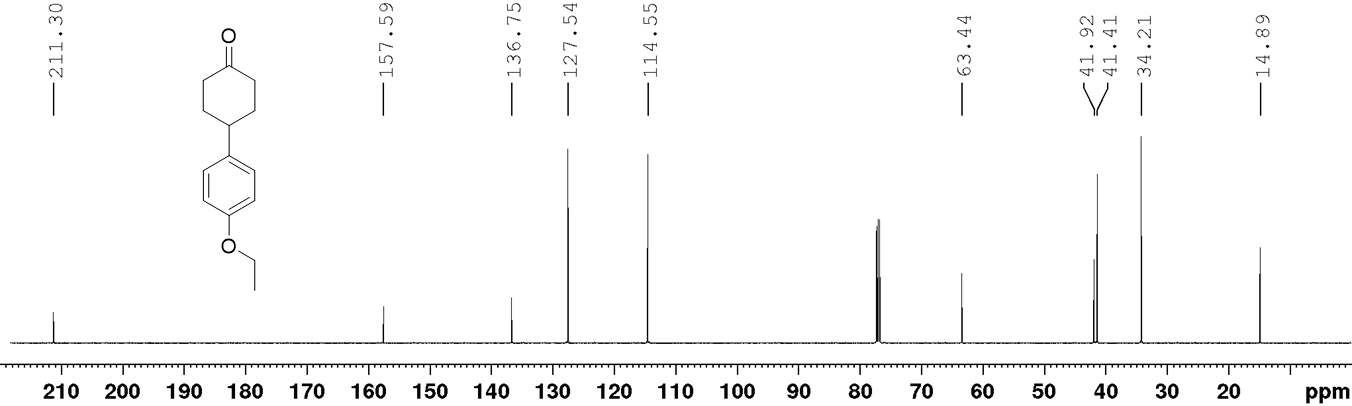


**Fig. S2**. ^13^C NMR spectrum of intermediate compound 4-(4-ethoxyphenyl)cyclohexan-1-one


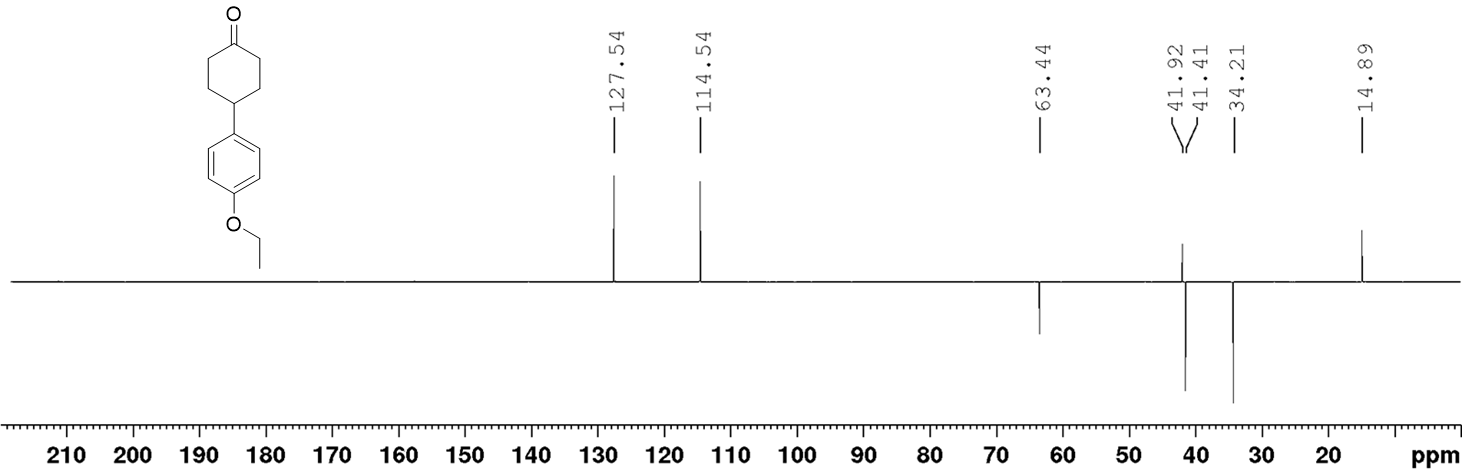


**Fig. S3**. ^13^C DEPT 135 NMR spectrum of intermediate compound 4-(4-ethoxyphenyl)cyclohexan-1-one


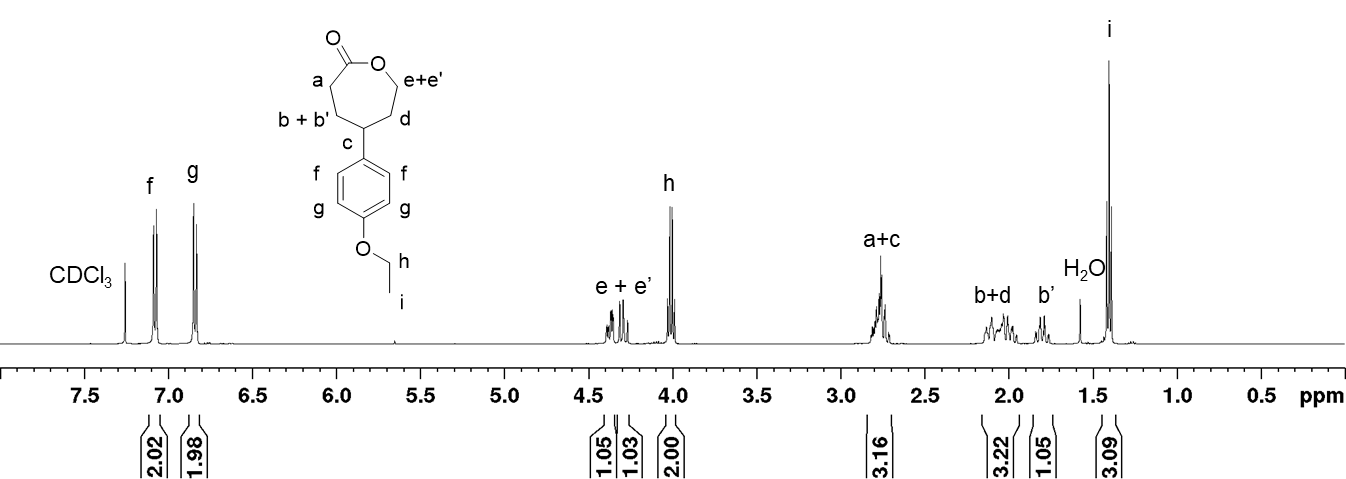


**Fig. S4**. ^1^H NMR spectrum of monomer γ-4-ethoxyphenyl ε-caprolactone (γ-EtOPhCL)
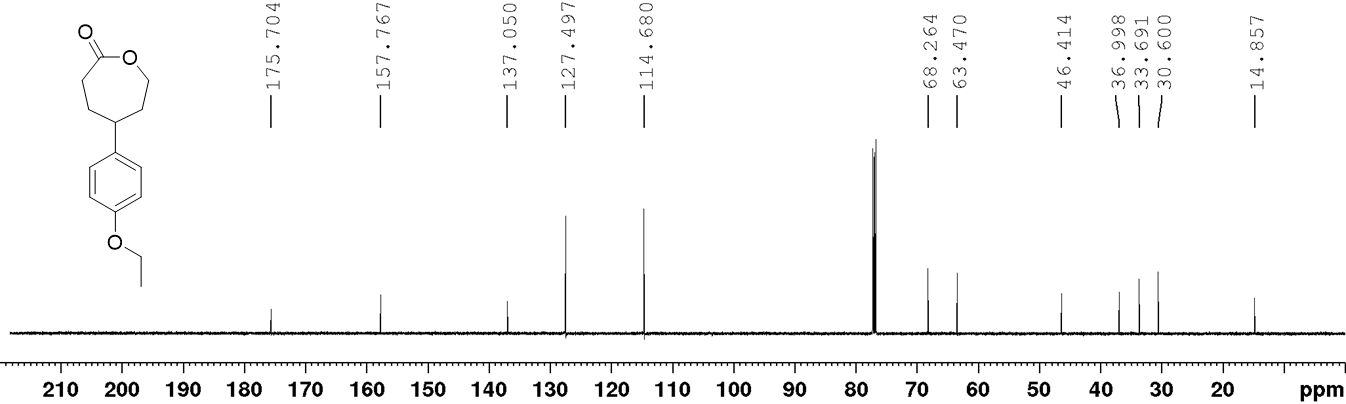


**Fig. S5**. 13C NMR spectrum of monomer γ-4-ethoxyphenyl ε-caprolactone (γ-EtOPhCL)


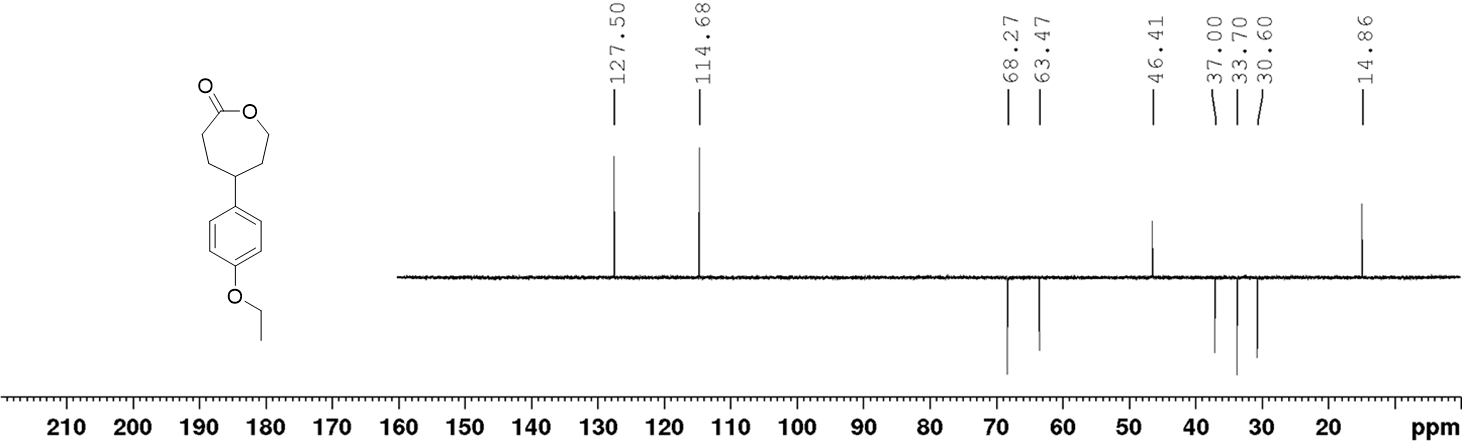


**Fig. S6**. ^13^C DEPT 135 NMR spectrum of monomer γ-4-ethoxyphenyl ε-caprolactone (γ-EtOPhCL)


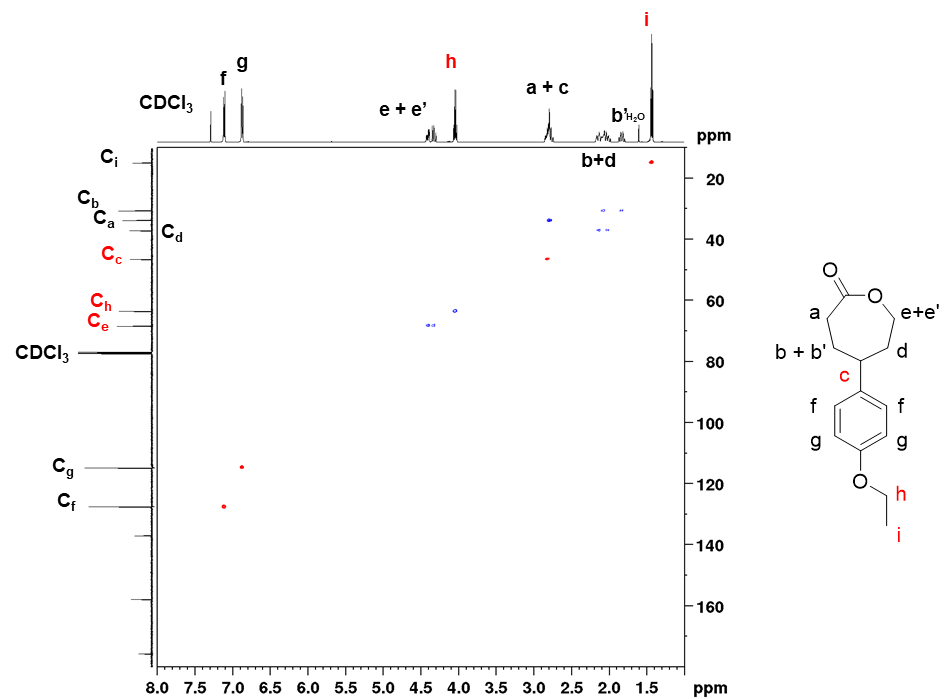


**Fig. S7**. HSQC NMR spectrum of monomer γ-4-ethoxyphenyl ε-caprolactone (γ-EtOPhCL)


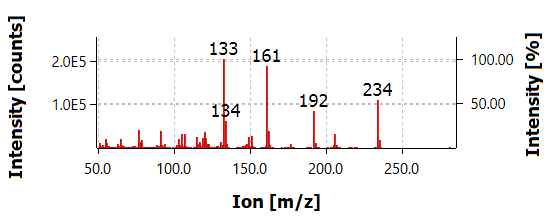


**Fig. S8**. GC-MS of monomer γ-4-ethoxyphenyl ε-caprolactone (γ-EtOPhCL)


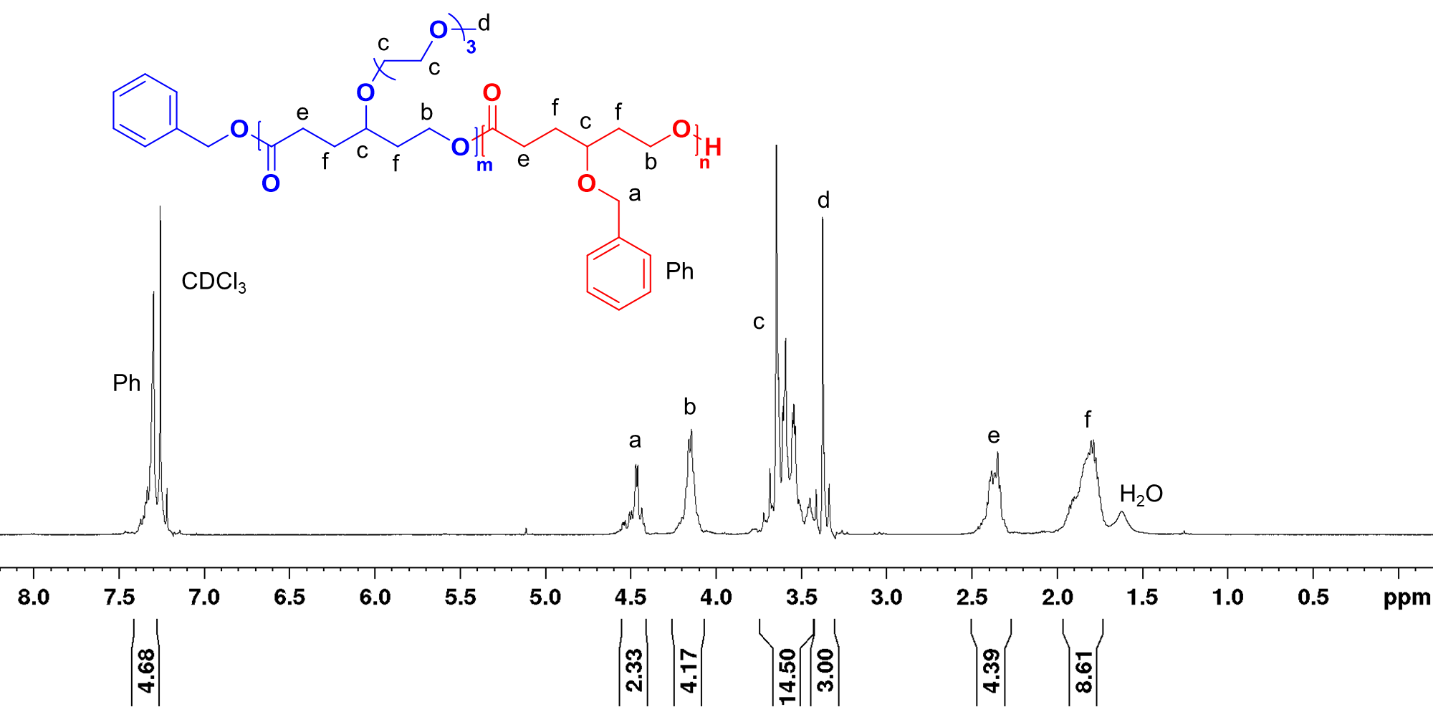


**Fig. S9**. ^1^H NMR spectrum of amphiphilic diblock poly{γ-2-[2-(2-methoxyethoxy)ethoxy]ethoxy-ε-caprolactone}-*b*-poly(γ-benzyloxy-ε-caprolactone) (PME_3_CL-*b*-PBnCL)


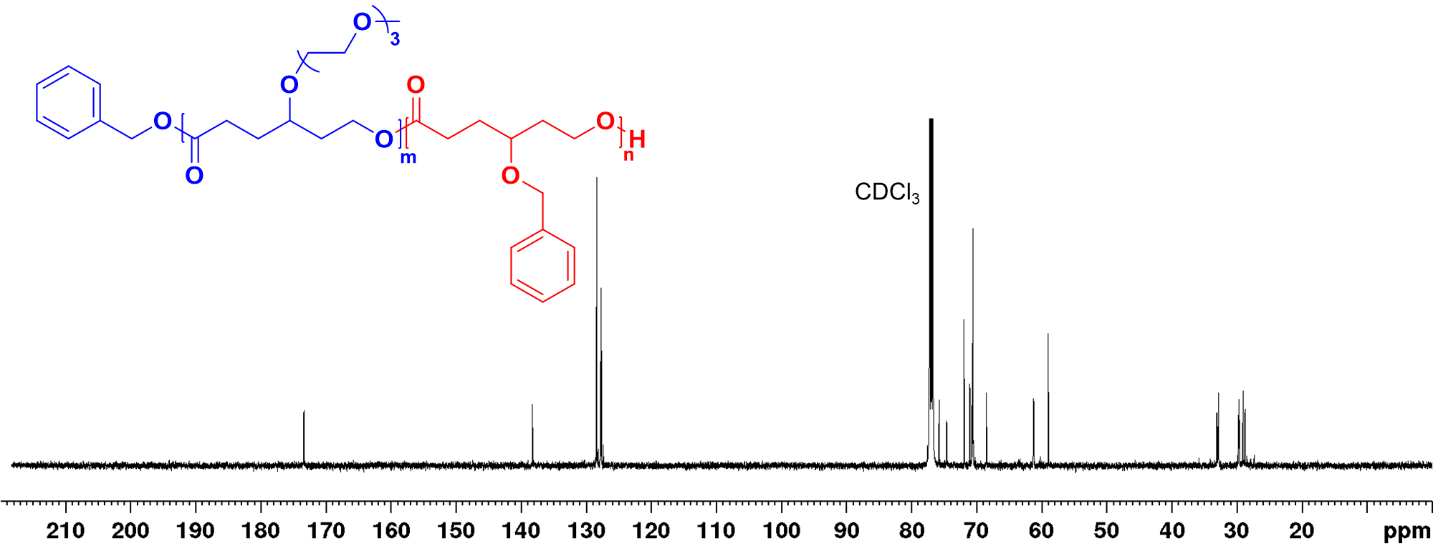


**Fig. S10**. ^13^C NMR spectrum of amphiphilic diblock copolymer poly{γ-2-[2-(2-methoxyethoxy)ethoxy]ethoxy-ε-caprolactone}-*b*-poly(γ-benzyloxy-ε-caprolactone) (PME_3_CL-*b*-PBnCL)


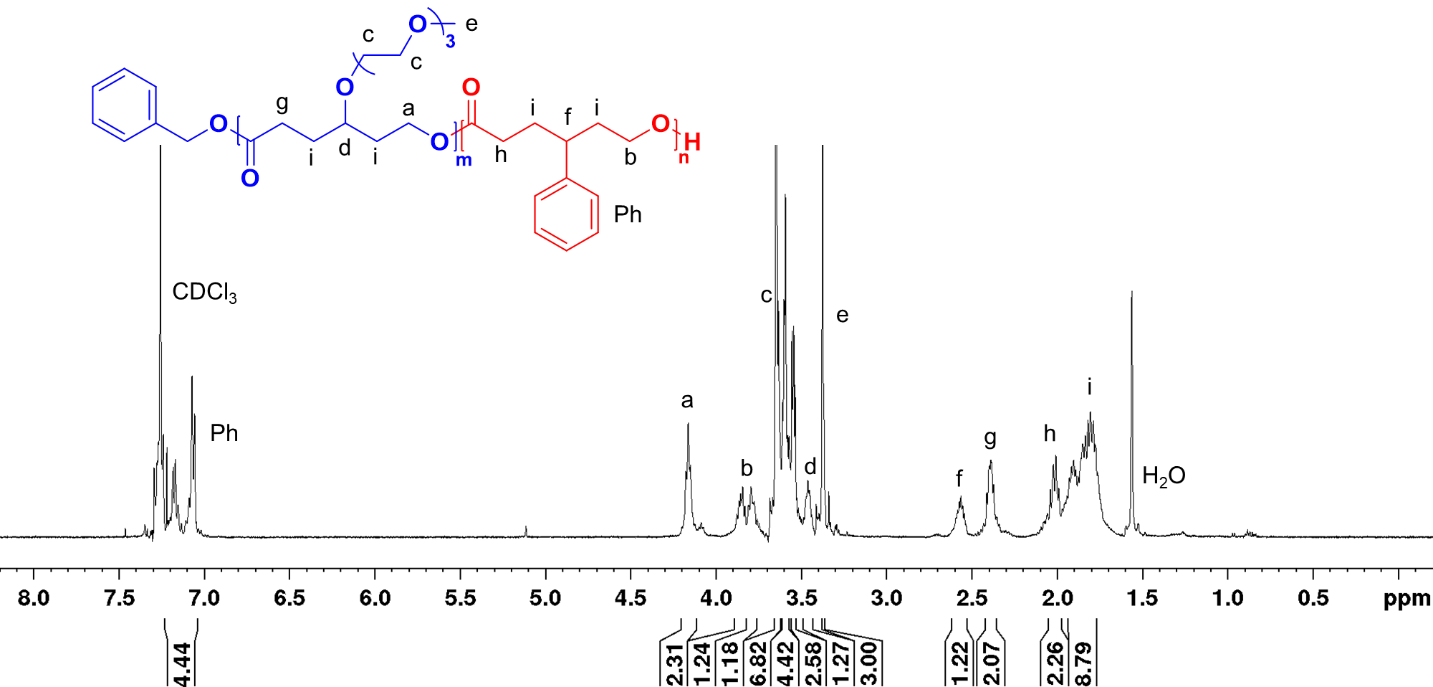


**Fig. S11**. ^1^H NMR spectrum of amphiphilic diblock copolymer poly{γ-2-[2-(2-methoxyethoxy)ethoxy]ethoxy-ε-caprolactone}-*b*-poly(γ-phenyl-ε-caprolactone) (PME_3_CL-*b*-PPhCL)


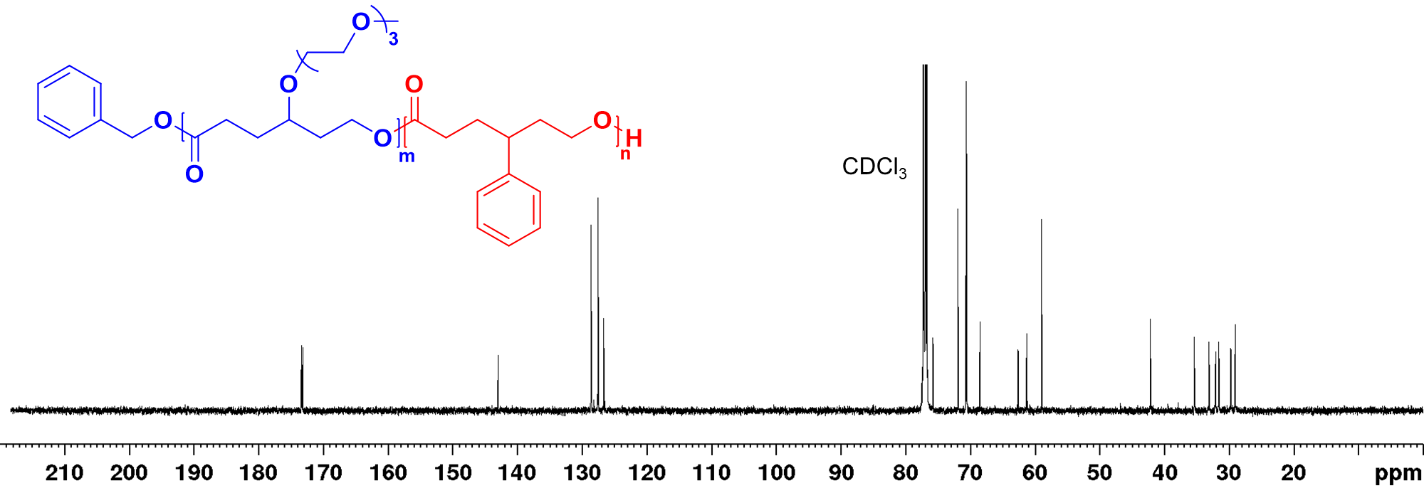


**Fig. S12**. ^13^C NMR spectrum of amphiphilic diblock copolymer poly{γ-2-[2-(2-methoxyethoxy)ethoxy]ethoxy-ε-caprolactone}-*b*-poly(γ-phenyl-ε-caprolactone) (PME_3_CL-*b*-PPhCL)


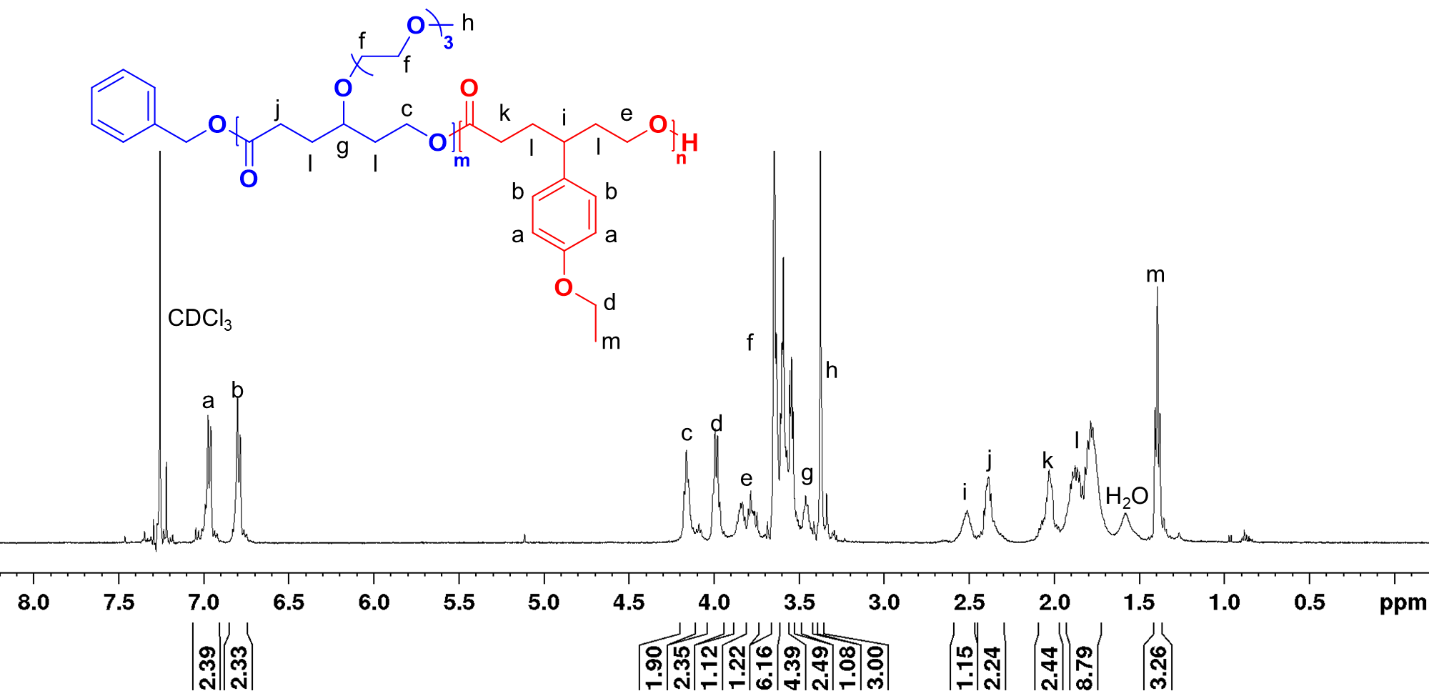


**Fig. S13**. ^1^H NMR spectrum of amphiphilic diblock copolymer poly{γ-2-[2-(2-methoxyethoxy)ethoxy]ethoxy-ε-caprolactone}-*b*-poly(γ-(*4*-ethoxylphenyl)-ε-caprolactone) (PME_3_CL-*b*-PEtOPhCL)


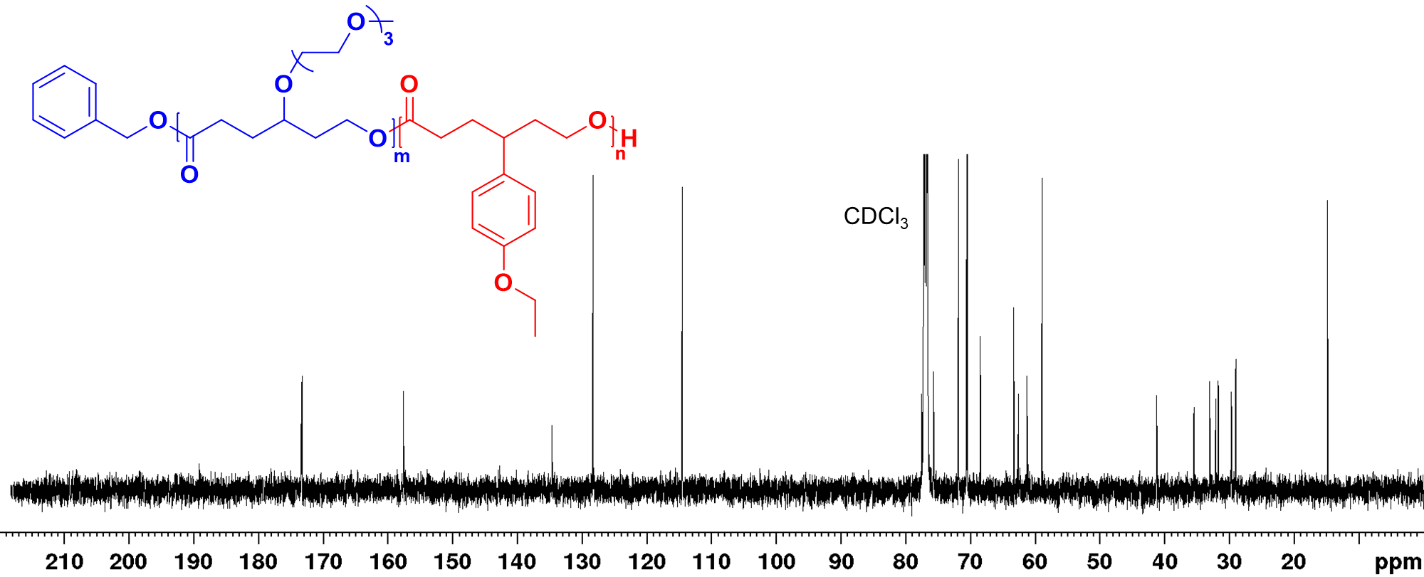


**Fig. S14**. ^13^C NMR spectrum of amphiphilic diblock copolymer poly{γ-2-[2-(2-methoxyethoxy)ethoxy]ethoxy-ε-caprolactone}-*b*-poly(γ-(*4*-ethoxylphenyl)-ε-caprolactone) (PME_3_CL-*b*-PEtOPhCL)

**Fig. S15**. GPC spectra of amphiphilic diblock copolymers PME_3_CL-*b*-PBnCL, PME_3_CL-*b*-PPhCL, and PME_3_CL-*b*-PEtOPhCL.

**Fig. S16**. DSC of amphiphilic diblock copolymers PME_3_CL-*b*-PBnCL, PME_3_CL-*b*-PPhCL, and PME_3_CL-*b*-PEtOPhCL.

**REFERENCES**

1. Hao, J.; Cheng, Y.; Ranatunga, R. J. K. U.; Senevirathne, S.; Biewer, M. C.; Nielsen, S. O.; Wang, Q.; Stefan, M. C., A Combined Experimental and Computational Study of the Substituent Effect on Micellar Behavior of γ-Substituted Thermoresponsive Amphiphilic Poly(ε-caprolactone)s. *Macromolecules (Washington, DC, U. S.)* **2013,** *46* (12), 4829-4838.

2. Soltantabar, P.; Calubaquib, E. L.; Mostafavi, E.; Biewer, M. C.; Stefan, M. C., Enhancement of Loading Efficiency by Coloading of Doxorubicin and Quercetin in Thermoresponsive Polymeric Micelles. *Biomacromolecules* **2020,** *21* (4), 1427-1436.

3. Deng, X.; Yuan, M.; Cao, X.; Li, X., Polymerization of Lactides and Lactones, 8. Study on the Ring‐Opening Polymerization of 3‐Phenyl‐ε‐caprolactone and 5‐Phenyl‐ε‐caprolactone. *Macromolecular Chemistry and Physics* **2001,** *202* (11), 2417-2424.
